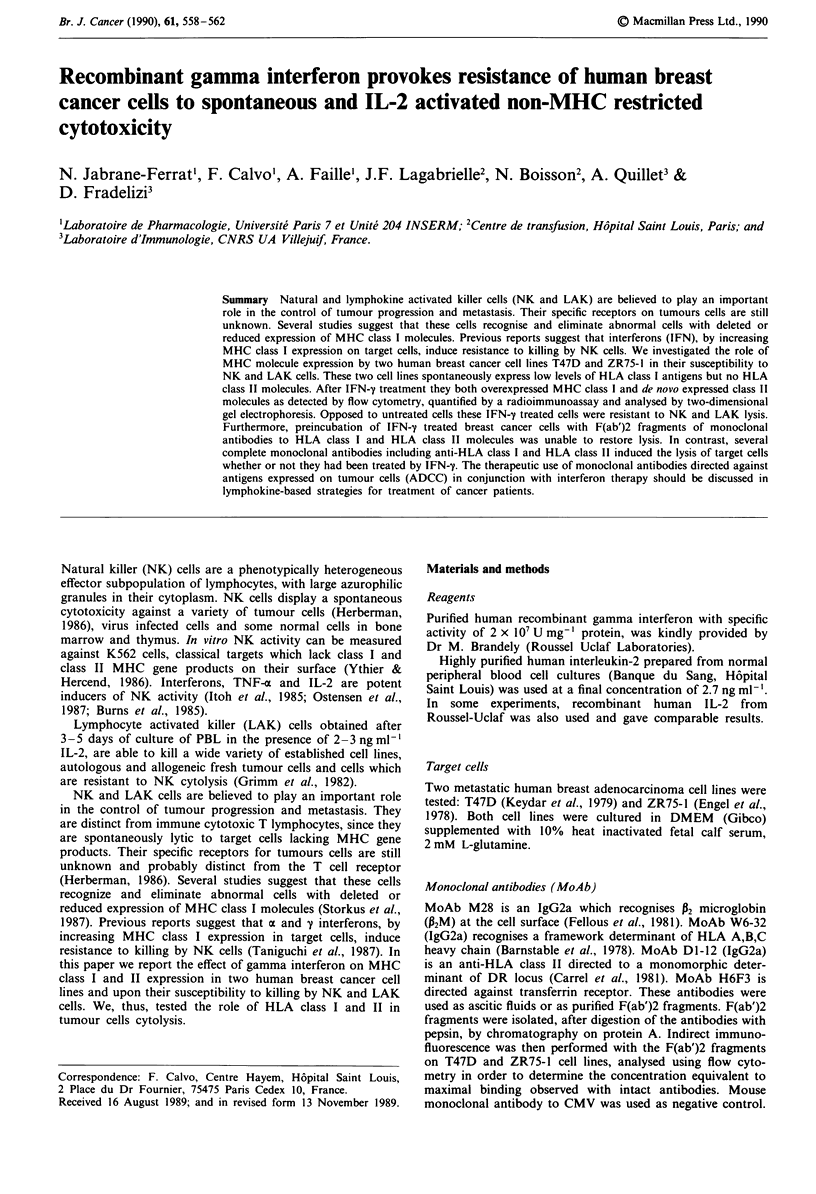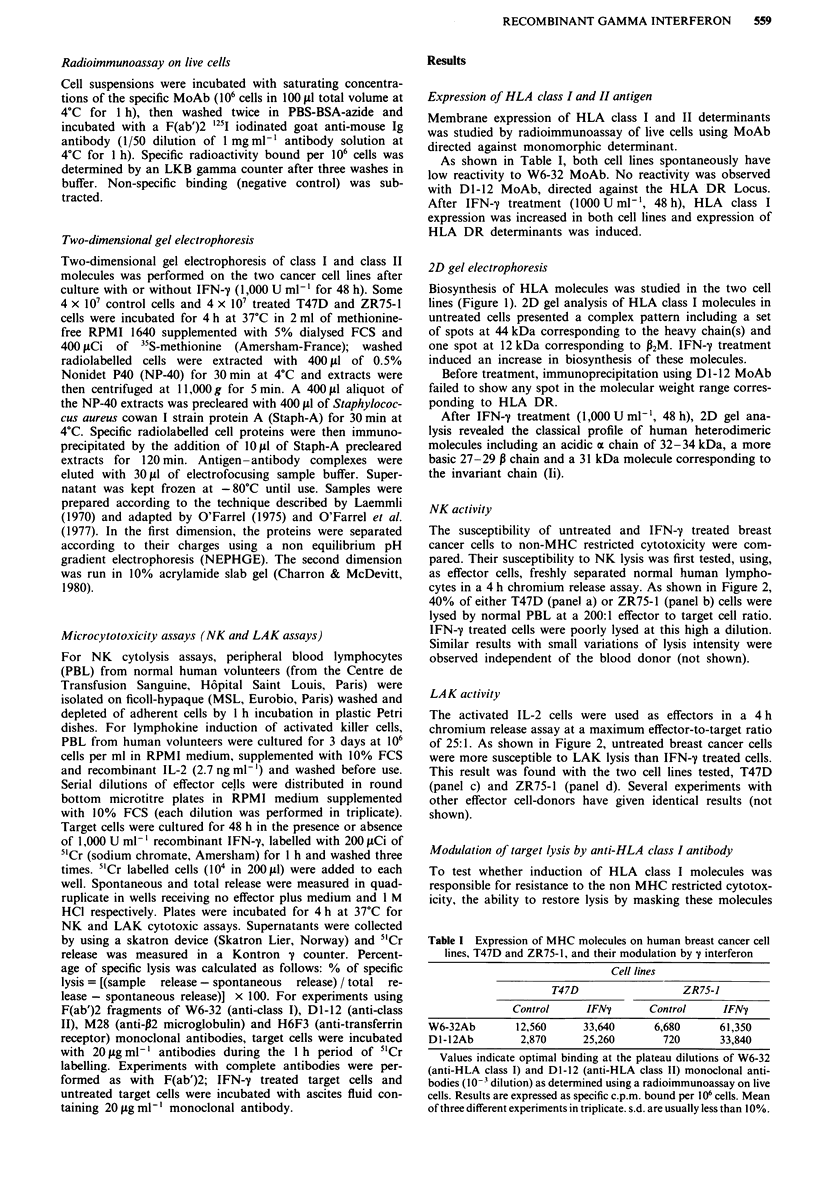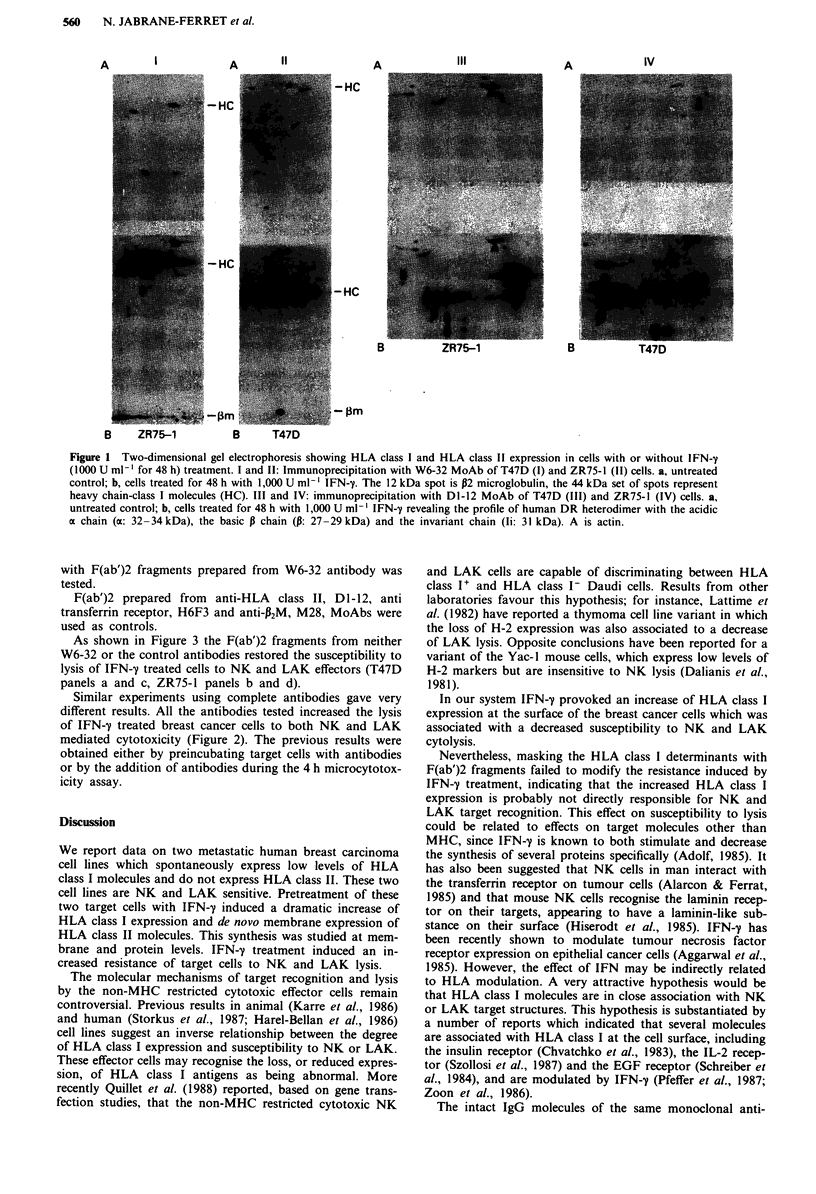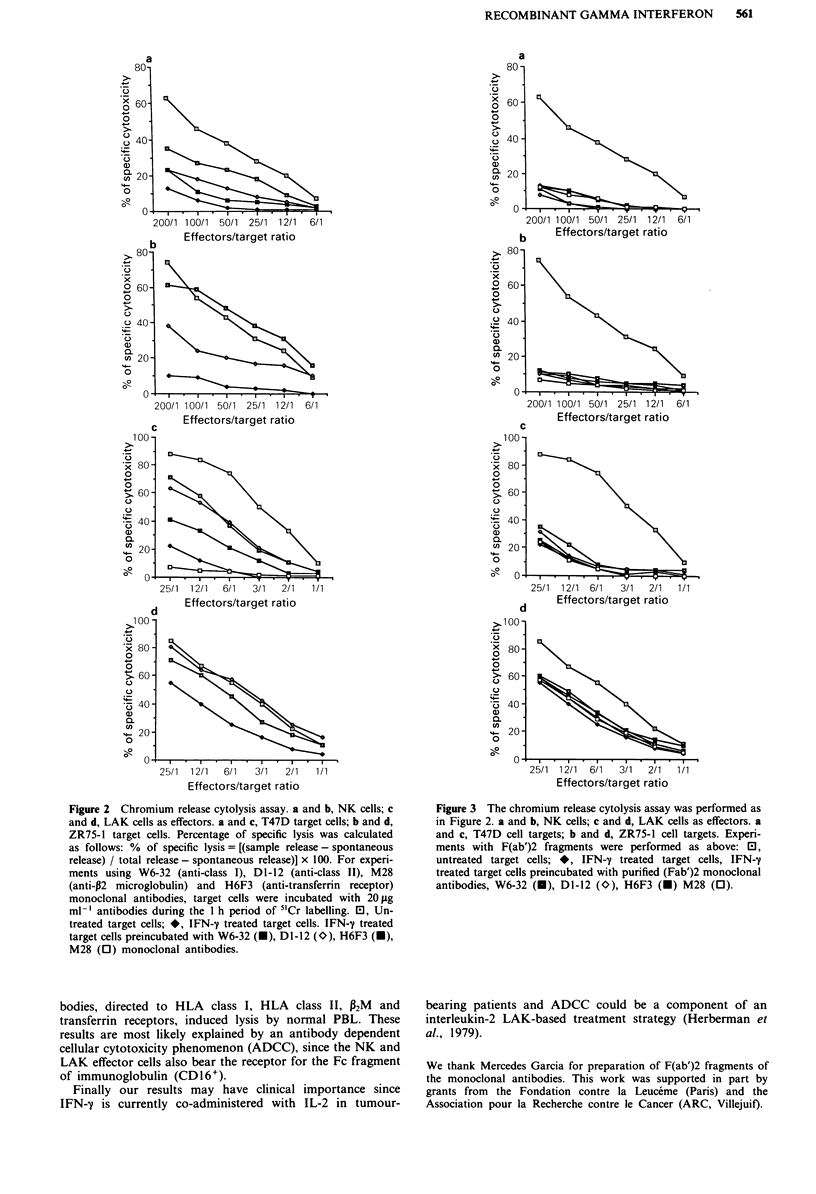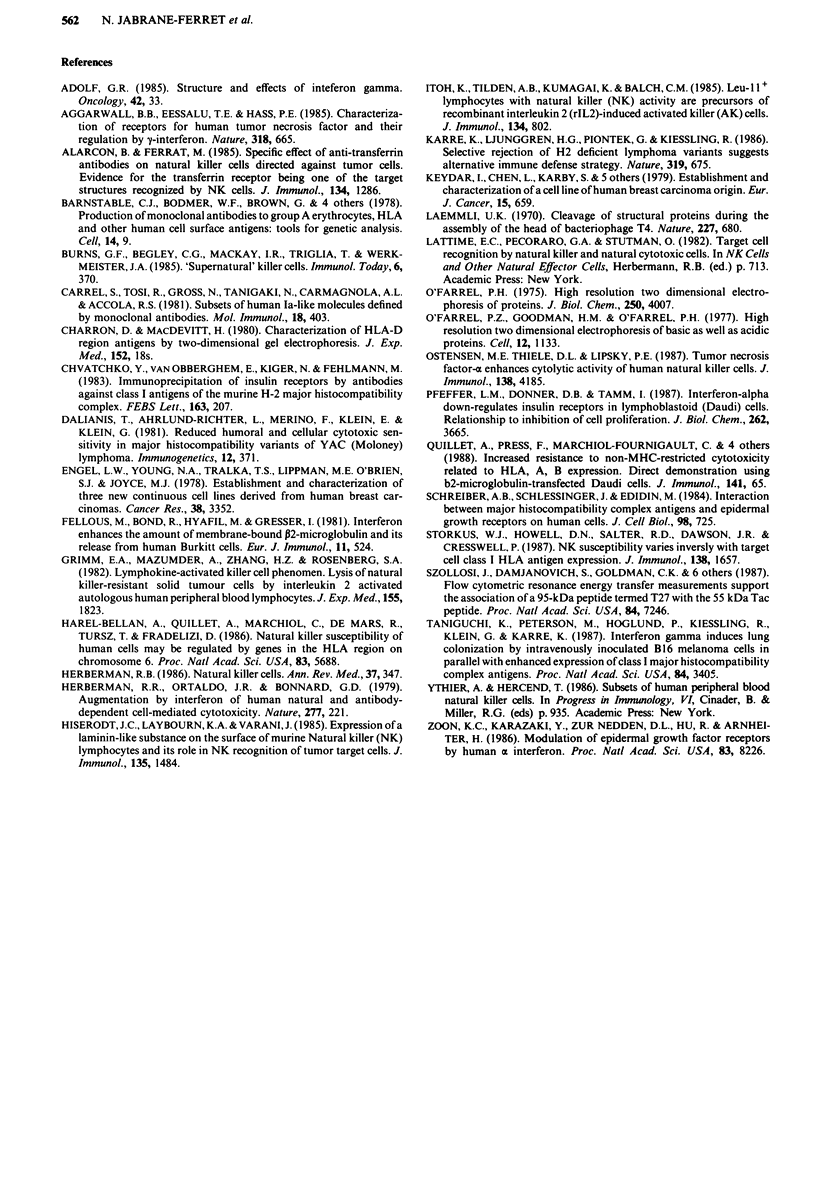# Recombinant gamma interferon provokes resistance of human breast cancer cells to spontaneous and IL-2 activated non-MHC restricted cytotoxicity.

**DOI:** 10.1038/bjc.1990.125

**Published:** 1990-04

**Authors:** N. Jabrane-Ferrat, F. Calvo, A. Faille, J. F. Lagabrielle, N. Boisson, A. Quillet, D. Fradelizi

**Affiliations:** Laboratoire de Pharmacologie, Université Paris 7, France.

## Abstract

**Images:**


Br. J. Cancer (1990), 61, 558–562 © Macmillan Press Ltd., 1990

### Recombinant gamma interferon provokes resistance of human breast cancer cells to spontaneous and IL-2 activated non-MHC restricted cytotoxicity

N. Jabrane-Ferrat[1], F. Calvo[1], A. Faille[1], J.F. Lagabrielle[2], N. Boisson[2], A. Quillet[3] & D. Fradelizi[3]

[1]*Laboratoire de Pharmacologie, Université Paris 7 et Unité 204 INSERM;* [2]*Centre de transfusion, Hôpital Saint Louis, Paris; and* [3]*Laboratoire d'Immunologie, CNRS UA Villejuif, France.*

**Summary** Natural and lymphokine activated killer cells (NK and LAK) are believed to play an important role in the control of tumour progression and metastasis. Their specific receptors on tumours cells are still unknown. Several studies suggest that these cells recognise and eliminate abnormal cells with deleted or reduced expression of MHC class I molecules. Previous reports suggest that interferons (IFN), by increasing MHC class I expression on target cells, induce resistance to killing by NK cells. We investigated the role of MHC molecule expression by two human breast cancer cell lines T47D and ZR75-1 in their susceptibility to NK and LAK cells. These two cell lines spontaneously express low levels of HLA class I antigens but no HLA class II molecules. After IFN-γ treatment they both overexpressed MHC class I and *de novo* expressed class II molecules as detected by flow cytometry, quantified by a radioimmunoassay and analysed by two-dimensional gel electrophoresis. Opposed to untreated cells these IFN-γ treated cells were resistant to NK and LAK lysis. Furthermore, preincubation of IFN-γ treated breast cancer cells with F(ab')2 fragments of monoclonal antibodies to HLA class I and HLA class II molecules was unable to restore lysis. In contrast, several complete monoclonal antibodies including anti-HLA class I and HLA class II induced the lysis of target cells whether or not they had been treated by IFN-γ. The therapeutic use of monoclonal antibodies directed against antigens expressed on tumour cells (ADCC) in conjunction with interferon therapy should be discussed in lymphokine-based strategies for treatment of cancer patients.

Natural killer (NK) cells are a phenotypically heterogeneous effector subpopulation of lymphocytes, with large azurophilic granules in their cytoplasm. NK cells display a spontaneous cytotoxicity against a variety of tumour cells (Herberman, 1986), virus infected cells and some normal cells in bone marrow and thymus. *In vitro* NK activity can be measured against K562 cells, classical targets which lack class I and class II MHC gene products on their surface (Ythier & Hercend, 1986). Interferons, TNF-α and IL-2 are potent inducers of NK activity (Itoh *et al.*, 1985; Ostensen *et al.*, 1987; Burns *et al.*, 1985).

Lymphocyte activated killer (LAK) cells obtained after 3–5 days of culture of PBL in the presence of 2–3 ng ml$^{-1}$ IL-2, are able to kill a wide variety of established cell lines, autologous and allogeneic fresh tumour cells and cells which are resistant to NK cytolysis (Grimm *et al.*, 1982).

NK and LAK cells are believed to play an important role in the control of tumour progression and metastasis. They are distinct from immune cytotoxic T lymphocytes, since they are spontaneously lytic to target cells lacking MHC gene products. Their specific receptors for tumours cells are still unknown and probably distinct from the T cell receptor (Herberman, 1986). Several studies suggest that these cells recognize and eliminate abnormal cells with deleted or reduced expression of MHC class I molecules (Storkus *et al.*, 1987). Previous reports suggest that α and γ interferons, by increasing MHC class I expression in target cells, induce resistance to killing by NK cells (Taniguchi *et al.*, 1987). In this paper we report the effect of gamma interferon on MHC class I and II expression in two human breast cancer cell lines and upon their susceptibility to killing by NK and LAK cells. We, thus, tested the role of HLA class I and II in tumour cells cytolysis.

Correspondence: F. Calvo, Centre Hayem, Hôpital Saint Louis, 2 Place du Dr Fournier, 75475 Paris Cedex 10, France.
Received 16 August 1989; and in revised form 13 November 1989.

### Materials and methods

### *Reagents*

Purified human recombinant gamma interferon with specific activity of $2 \times 10^7$ U mg$^{-1}$ protein, was kindly provided by Dr M. Brandely (Roussel Uclaf Laboratories).

Highly purified human interleukin-2 prepared from normal peripheral blood cell cultures (Banque du Sang, Hôpital Saint Louis) was used at a final concentration of 2.7 ng ml$^{-1}$. In some experiments, recombinant human IL-2 from Roussel-Uclaf was also used and gave comparable results.

### *Target cells*

Two metastatic human breast adenocarcinoma cell lines were tested: T47D (Keydar *et al.*, 1979) and ZR75-1 (Engel *et al.*, 1978). Both cell lines were cultured in DMEM (Gibco) supplemented with 10% heat inactivated fetal calf serum, 2 mM L-glutamine.

### *Monoclonal antibodies (MoAb)*

MoAb M28 is an IgG2a which recognises $\beta_2$ microglobin ($\beta_2$M) at the cell surface (Fellous *et al.*, 1981). MoAb W6-32 (IgG2a) recognises a framework determinant of HLA A,B,C heavy chain (Barnstable *et al.*, 1978). MoAb D1-12 (IgG2a) is an anti-HLA class II directed to a monomorphic determinant of DR locus (Carrel *et al.*, 1981). MoAb H6F3 is directed against transferrin receptor. These antibodies were used as ascitic fluids or as purified F(ab')2 fragments. F(ab')2 fragments were isolated, after digestion of the antibodies with pepsin, by chromatography on protein A. Indirect immunofluorescence was then performed with the F(ab')2 fragments on T47D and ZR75-1 cell lines, analysed using flow cytometry in order to determine the concentration equivalent to maximal binding observed with intact antibodies. Mouse monoclonal antibody to CMV was used as negative control.

*Radioimmunoassay on live cells*

Cell suspensions were incubated with saturating concentrations of the specific MoAb ($10^6$ cells in 100 µl total volume at 4°C for 1 h), then washed twice in PBS-BSA-azide and incubated with a F(ab')2 $^{125}$I iodinated goat anti-mouse Ig antibody (1/50 dilution of 1 mg ml$^{-1}$ antibody solution at 4°C for 1 h). Specific radioactivity bound per $10^6$ cells was determined by an LKB gamma counter after three washes in buffer. Non-specific binding (negative control) was subtracted.

*Two-dimensional gel electrophoresis*

Two-dimensional gel electrophoresis of class I and class II molecules was performed on the two cancer cell lines after culture with or without IFN-γ (1,000 U ml$^{-1}$ for 48 h). Some $4 \times 10^7$ control cells and $4 \times 10^7$ treated T47D and ZR75-1 cells were incubated for 4 h at 37°C in 2 ml of methionine-free RPMI 1640 supplemented with 5% dialysed FCS and 400 µCi of $^{35}$S-methionine (Amersham-France); washed radiolabelled cells were extracted with 400 µl of 0.5% Nonidet P40 (NP-40) for 30 min at 4°C and extracts were then centrifuged at 11,000 *g* for 5 min. A 400 µl aliquot of the NP-40 extracts was precleared with 400 µl of *Staphylococcus aureus* cowan I strain protein A (Staph-A) for 30 min at 4°C. Specific radiolabelled cell proteins were then immunoprecipitated by the addition of 10 µl of Staph-A precleared extracts for 120 min. Antigen–antibody complexes were eluted with 30 µl of electrofocusing sample buffer. Supernatant was kept frozen at −80°C until use. Samples were prepared according to the technique described by Laemmli (1970) and adapted by O'Farrel (1975) and O'Farrel *et al.* (1977). In the first dimension, the proteins were separated according to their charges using a non equilibrium pH gradient electrophoresis (NEPHGE). The second dimension was run in 10% acrylamide slab gel (Charron & McDevitt, 1980).

*Microcytotoxicity assays (NK and LAK assays)*

For NK cytolysis assays, peripheral blood lymphocytes (PBL) from normal human volunteers (from the Centre de Transfusion Sanguine, Hôpital Saint Louis, Paris) were isolated on ficoll-hypaque (MSL, Eurobio, Paris) washed and depleted of adherent cells by 1 h incubation in plastic Petri dishes. For lymphokine induction of activated killer cells, PBL from human volunteers were cultured for 3 days at $10^6$ cells per ml in RPMI medium, supplemented with 10% FCS and recombinant IL-2 (2.7 ng ml$^{-1}$) and washed before use. Serial dilutions of effector cells were distributed in round bottom microtitre plates in RPMI medium supplemented with 10% FCS (each dilution was performed in triplicate). Target cells were cultured for 48 h in the presence or absence of 1,000 U ml$^{-1}$ recombinant IFN-γ, labelled with 200 µCi of $^{51}$Cr (sodium chromate, Amersham) for 1 h and washed three times. $^{51}$Cr labelled cells ($10^4$ in 200 µl) were added to each well. Spontaneous and total release were measured in quadruplicate in wells receiving no effector plus medium and 1 M HCl respectively. Plates were incubated for 4 h at 37°C for NK and LAK cytotoxic assays. Supernatants were collected by using a skatron device (Skatron Lier, Norway) and $^{51}$Cr release was measured in a Kontron γ counter. Percentage of specific lysis was calculated as follows: % of specific lysis = [(sample release − spontaneous release) / total release − spontaneous release)] × 100. For experiments using F(ab')2 fragments of W6-32 (anti-class I), D1-12 (anti-class II), M28 (anti-β2 microglobulin) and H6F3 (anti-transferrin receptor) monoclonal antibodies, target cells were incubated with 20 µg ml$^{-1}$ antibodies during the 1 h period of $^{51}$Cr labelling. Experiments with complete antibodies were performed as with F(ab')2; IFN-γ treated target cells and untreated target cells were incubated with ascites fluid containing 20 µg ml$^{-1}$ monoclonal antibody.

### Results

*Expression of HLA class I and II antigen*

Membrane expression of HLA class I and II determinants was studied by radioimmunoassay of live cells using MoAb directed against monomorphic determinant.

As shown in Table I, both cell lines spontaneously have low reactivity to W6-32 MoAb. No reactivity was observed with D1-12 MoAb, directed against the HLA DR Locus. After IFN-γ treatment (1000 U ml$^{-1}$, 48 h), HLA class I expression was increased in both cell lines and expression of HLA DR determinants was induced.

*2D gel electrophoresis*

Biosynthesis of HLA molecules was studied in the two cell lines (Figure 1). 2D gel analysis of HLA class I molecules in untreated cells presented a complex pattern including a set of spots at 44 kDa corresponding to the heavy chain(s) and one spot at 12 kDa corresponding to $\beta_2$M. IFN-γ treatment induced an increase in biosynthesis of these molecules.

Before treatment, immunoprecipitation using D1-12 MoAb failed to show any spot in the molecular weight range corresponding to HLA DR.

After IFN-γ treatment (1,000 U ml$^{-1}$, 48 h), 2D gel analysis revealed the classical profile of human heterodimeric molecules including an acidic α chain of 32–34 kDa, a more basic 27–29 β chain and a 31 kDa molecule corresponding to the invariant chain (Ii).

*NK activity*

The susceptibility of untreated and IFN-γ treated breast cancer cells to non-MHC restricted cytotoxicity were compared. Their susceptibility to NK lysis was first tested, using, as effector cells, freshly separated normal human lymphocytes in a 4 h chromium release assay. As shown in Figure 2, 40% of either T47D (panel a) or ZR75-1 (panel b) cells were lysed by normal PBL at a 200:1 effector to target cell ratio. IFN-γ treated cells were poorly lysed at this high a dilution. Similar results with small variations of lysis intensity were observed independent of the blood donor (not shown).

*LAK activity*

The activated IL-2 cells were used as effectors in a 4 h chromium release assay at a maximum effector-to-target ratio of 25:1. As shown in Figure 2, untreated breast cancer cells were more susceptible to LAK lysis than IFN-γ treated cells. This result was found with the two cell lines tested, T47D (panel c) and ZR75-1 (panel d). Several experiments with other effector cell-donors have given identical results (not shown).

*Modulation of target lysis by anti-HLA class I antibody*

To test whether induction of HLA class I molecules was responsible for resistance to the non MHC restricted cytotoxicity, the ability to restore lysis by masking these molecules

**Table I** Expression of MHC molecules on human breast cancer cell lines, T47D and ZR75-1, and their modulation by γ interferon

| | Cell lines | | | |
|---|---|---|---|---|
| | T47D | | ZR75-1 | |
| | Control | IFNγ | Control | IFNγ |
| W6-32Ab | 12,560 | 33,640 | 6,680 | 61,350 |
| D1-12Ab | 2,870 | 25,260 | 720 | 33,840 |

Values indicate optimal binding at the plateau dilutions of W6-32 (anti-HLA class I) and D1-12 (anti-HLA class II) monoclonal antibodies ($10^{-3}$ dilution) as determined using a radioimmunoassay on live cells. Results are expressed as specific c.p.m. bound per $10^6$ cells. Mean of three different experiments in triplicate. s.d. are usually less than 10%.

**Figure 1** Two-dimensional gel electrophoresis showing HLA class I and HLA class II expression in cells with or without IFN-γ (1000 U $ml^{-1}$ for 48 h) treatment. I and II: Immunoprecipitation with W6-32 MoAb of T47D (I) and ZR75-1 (II) cells. **a**, untreated control; **b**, cells treated for 48 h with 1,000 U $ml^{-1}$ IFN-γ. The 12 kDa spot is β2 microglobulin, the 44 kDa set of spots represent heavy chain-class I molecules (HC). III and IV: immunoprecipitation with D1-12 MoAb of T47D (III) and ZR75-1 (IV) cells. **a**, untreated control; **b**, cells treated for 48 h with 1,000 U $ml^{-1}$ IFN-γ revealing the profile of human DR heterodimer with the acidic α chain (α: 32–34 kDa), the basic β chain (β: 27–29 kDa) and the invariant chain (Ii: 31 kDa). A is actin.

with F(ab')2 fragments prepared from W6-32 antibody was tested.

F(ab')2 prepared from anti-HLA class II, D1-12, anti transferrin receptor, H6F3 and anti-$\beta_2$M, M28, MoAbs were used as controls.

As shown in Figure 3 the F(ab')2 fragments from neither W6-32 or the control antibodies restored the susceptibility to lysis of IFN-γ treated cells to NK and LAK effectors (T47D panels a and c, ZR75-1 panels b and d).

Similar experiments using complete antibodies gave very different results. All the antibodies tested increased the lysis of IFN-γ treated breast cancer cells to both NK and LAK mediated cytotoxicity (Figure 2). The previous results were obtained either by preincubating target cells with antibodies or by the addition of antibodies during the 4 h microcytotoxicity assay.

### Discussion

We report data on two metastatic human breast carcinoma cell lines which spontaneously express low levels of HLA class I molecules and do not express HLA class II. These two cell lines are NK and LAK sensitive. Pretreatment of these two target cells with IFN-γ induced a dramatic increase of HLA class I expression and *de novo* membrane expression of HLA class II molecules. This synthesis was studied at membrane and protein levels. IFN-γ treatment induced an increased resistance of target cells to NK and LAK lysis.

The molecular mechanisms of target recognition and lysis by the non-MHC restricted cytotoxic effector cells remain controversial. Previous results in animal (Karre *et al.*, 1986) and human (Storkus *et al.*, 1987; Harel-Bellan *et al.*, 1986) cell lines suggest an inverse relationship between the degree of HLA class I expression and susceptibility to NK or LAK. These effector cells may recognise the loss, or reduced expression, of HLA class I antigens as being abnormal. More recently Quillet *et al.* (1988) reported, based on gene transfection studies, that the non-MHC restricted cytotoxic NK and LAK cells are capable of discriminating between HLA class $I^+$ and HLA class $I^-$ Daudi cells. Results from other laboratories favour this hypothesis; for instance, Lattime *et al.* (1982) have reported a thymoma cell line variant in which the loss of H-2 expression was also associated to a decrease of LAK lysis. Opposite conclusions have been reported for a variant of the Yac-1 mouse cells, which express low levels of H-2 markers but are insensitive to NK lysis (Dalianis *et al.*, 1981).

In our system IFN-γ provoked an increase of HLA class I expression at the surface of the breast cancer cells which was associated with a decreased susceptibility to NK and LAK cytolysis.

Nevertheless, masking the HLA class I determinants with F(ab')2 fragments failed to modify the resistance induced by IFN-γ treatment, indicating that the increased HLA class I expression is probably not directly responsible for NK and LAK target recognition. This effect on susceptibility to lysis could be related to effects on target molecules other than MHC, since IFN-γ is known to both stimulate and decrease the synthesis of several proteins specifically (Adolf, 1985). It has also been suggested that NK cells in man interact with the transferrin receptor on tumour cells (Alarcon & Ferrat, 1985) and that mouse NK cells recognise the laminin receptor on their targets, appearing to have a laminin-like substance on their surface (Hiserodt *et al.*, 1985). IFN-γ has been recently shown to modulate tumour necrosis factor receptor expression on epithelial cancer cells (Aggarwal *et al.*, 1985). However, the effect of IFN may be indirectly related to HLA modulation. A very attractive hypothesis would be that HLA class I molecules are in close association with NK or LAK target structures. This hypothesis is substantiated by a number of reports which indicated that several molecules are associated with HLA class I at the cell surface, including the insulin receptor (Chvatchko *et al.*, 1983), the IL-2 receptor (Szollosi *et al.*, 1987) and the EGF receptor (Schreiber *et al.*, 1984), and are modulated by IFN-γ (Pfeffer *et al.*, 1987; Zoon *et al.*, 1986).

The intact IgG molecules of the same monoclonal anti-

**Figure 2** Chromium release cytolysis assay. **a** and **b**, NK cells; **c** and **d**, LAK cells as effectors. **a** and **c**, T47D target cells; **b** and **d**, ZR75-1 target cells. Percentage of specific lysis was calculated as follows: % of specific lysis = [(sample release − spontaneous release) / total release − spontaneous release)] × 100. For experiments using W6-32 (anti-class I), D1-12 (anti-class II), M28 (anti-$\beta 2$ microglobulin) and H6F3 (anti-transferrin receptor) monoclonal antibodies, target cells were incubated with 20 $\mu$g $ml^{-1}$ antibodies during the 1 h period of $^{51}Cr$ labelling. ▣, Untreated target cells; ◆, IFN-γ treated target cells. IFN-γ treated target cells preincubated with W6-32 (■), D1-12 (◇), H6F3 (■), M28 (□) monoclonal antibodies.

**Figure 3** The chromium release cytolysis assay was performed as in Figure 2. **a** and **b**, NK cells; **c** and **d**, LAK cells as effectors. **a** and **c**, T47D cell targets; **b** and **d**, ZR75-1 cell targets. Experiments with F(ab')2 fragments were performed as above: ▣, untreated target cells; ◆, IFN-γ treated target cells, IFN-γ treated target cells preincubated with purified (Fab')2 monoclonal antibodies, W6-32 (■), D1-12 (◇), H6F3 (■) M28 (□).

bodies, directed to HLA class I, HLA class II, $\beta_2 M$ and transferrin receptors, induced lysis by normal PBL. These results are most likely explained by an antibody dependent cellular cytotoxicity phenomenon (ADCC), since the NK and LAK effector cells also bear the receptor for the Fc fragment of immunoglobulin ($CD16^+$).

Finally our results may have clinical importance since IFN-γ is currently co-administered with IL-2 in tumour-bearing patients and ADCC could be a component of an interleukin-2 LAK-based treatment strategy (Herberman *et al.*, 1979).

We thank Mercedes Garcia for preparation of F(ab')2 fragments of the monoclonal antibodies. This work was supported in part by grants from the Fondation contre la Leucéme (Paris) and the Association pour la Recherche contre le Cancer (ARC, Villejuif).

### References

ADOLF, G.R. (1985). Structure and effects of inteferon gamma. *Oncology*, **42**, 33.

AGGARWALL, B.B., EESSALU, T.E. & HASS, P.E. (1985). Characterization of receptors for human tumor necrosis factor and their regulation by γ-interferon. *Nature*, **318**, 665.

ALARCON, B. & FERRAT, M. (1985). Specific effect of anti-transferrin antibodies on natural killer cells directed against tumor cells. Evidence for the transferrin receptor being one of the target structures recognized by NK cells. *J. Immunol.*, **134**, 1286.

BARNSTABLE, C.J., BODMER, W.F., BROWN, G. & 4 others (1978). Production of monoclonal antibodies to group A erythrocytes, HLA and other human cell surface antigens: tools for genetic analysis. *Cell*, **14**, 9.

BURNS, G.F., BEGLEY, C.G., MACKAY, I.R., TRIGLIA, T. & WERKMEISTER, J.A. (1985). 'Supernatural' killer cells. *Immunol. Today*, **6**, 370.

CARREL, S., TOSI, R., GROSS, N., TANIGAKI, N., CARMAGNOLA, A.L. & ACCOLA, R.S. (1981). Subsets of human Ia-like molecules defined by monoclonal antibodies. *Mol. Immunol.*, **18**, 403.

CHARRON, D. & MACDEVITT, H. (1980). Characterization of HLA-D region antigens by two-dimensional gel electrophoresis. *J. Exp. Med.*, **152**, 18s.

CHVATCHKO, Y., VAN OBBERGHEM, E., KIGER, N. & FEHLMANN, M. (1983). Immunoprecipitation of insulin receptors by antibodies against class I antigens of the murine H-2 major histocompatibility complex. *FEBS Lett.*, **163**, 207.

DALIANIS, T., AHRLUND-RICHTER, L., MERINO, F., KLEIN, E. & KLEIN, G. (1981). Reduced humoral and cellular cytotoxic sensitivity in major histocompatibility variants of YAC (Moloney) lymphoma. *Immunogenetics*, **12**, 371.

ENGEL, L.W., YOUNG, N.A., TRALKA, T.S., LIPPMAN, M.E. O'BRIEN, S.J. & JOYCE, M.J. (1978). Establishment and characterization of three new continuous cell lines derived from human breast carcinomas. *Cancer Res.*, **38**, 3352.

FELLOUS, M., BOND, R., HYAFIL, M. & GRESSER, I. (1981). Interferon enhances the amount of membrane-bound β2-microglobulin and its release from human Burkitt cells. *Eur. J. Immunol.*, **11**, 524.

GRIMM, E.A., MAZUMDER, A., ZHANG, H.Z. & ROSENBERG, S.A. (1982). Lymphokine-activated killer cell phenomen. Lysis of natural killer-resistant solid tumour cells by interleukin 2 activated autologous human peripheral blood lymphocytes. *J. Exp. Med.*, **155**, 1823.

HAREL-BELLAN, A., QUILLET, A., MARCHIOL, C., DE MARS, R., TURSZ, T. & FRADELIZI, D. (1986). Natural killer susceptibility of human cells may be regulated by genes in the HLA region on chromosome 6. *Proc. Natl Acad. Sci. USA*, **83**, 5688.

HERBERMAN, R.B. (1986). Natural killer cells. *Ann. Rev. Med.*, **37**, 347.

HERBERMAN, R.R., ORTALDO, J.R. & BONNARD, G.D. (1979). Augmentation by interferon of human natural and antibody-dependent cell-mediated cytotoxicity. *Nature*, **277**, 221.

HISERODT, J.C., LAYBOURN, K.A. & VARANI, J. (1985). Expression of a laminin-like substance on the surface of murine Natural killer (NK) lymphocytes and its role in NK recognition of tumor target cells. *J. Immunol.*, **135**, 1484.

ITOH, K., TILDEN, A.B., KUMAGAI, K. & BALCH, C.M. (1985). Leu-11$^{+}$ lymphocytes with natural killer (NK) activity are precursors of recombinant interleukin 2 (rIL2)-induced activated killer (AK) cells. *J. Immunol.*, **134**, 802.

KARRE, K., LJUNGGREN, H.G., PIONTEK, G. & KIESSLING, R. (1986). Selective rejection of H2 deficient lymphoma variants suggests alternative immune defense strategy. *Nature*, **319**, 675.

KEYDAR, I., CHEN, L., KARBY, S. & 5 others (1979). Establishment and characterization of a cell line of human breast carcinoma origin. *Eur. J. Cancer*, **15**, 659.

LAEMMLI, U.K. (1970). Cleavage of structural proteins during the assembly of the head of bacteriophage T4. *Nature*, **227**, 680.

LATTIME, E.C., PECORARO, G.A. & STUTMAN, O. (1982). Target cell recognition by natural killer and natural cytotoxic cells. In *NK Cells and Other Natural Effector Cells*, Herbermann, R.B. (ed.) p. 713. Academic Press: New York.

O'FARREL, P.H. (1975). High resolution two dimensional electrophoresis of proteins. *J. Biol. Chem.*, **250**, 4007.

O'FARREL, P.Z., GOODMAN, H.M. & O'FARREL, P.H. (1977). High resolution two dimensional electrophoresis of basic as well as acidic proteins. *Cell*, **12**, 1133.

OSTENSEN, M.E. THIELE, D.L. & LIPSKY, P.E. (1987). Tumor necrosis factor-α enhances cytolytic activity of human natural killer cells. *J. Immunol.*, **138**, 4185.

PFEFFER, L.M., DONNER, D.B. & TAMM, I. (1987). Interferon-alpha down-regulates insulin receptors in lymphoblastoid (Daudi) cells. Relationship to inhibition of cell proliferation. *J. Biol. Chem.*, **262**, 3665.

QUILLET, A., PRESS, F., MARCHIOL-FOURNIGAULT, C. & 4 others (1988). Increased resistance to non-MHC-restricted cytotoxicity related to HLA, A, B expression. Direct demonstration using b2-microglobulin-transfected Daudi cells. *J. Immunol.*, **141**, 65.

SCHREIBER, A.B., SCHLESSINGER, J. & EDIDIN, M. (1984). Interaction between major histocompatibility complex antigens and epidermal growth receptors on human cells. *J. Cell Biol.*, **98**, 725.

STORKUS, W.J., HOWELL, D.N., SALTER, R.D., DAWSON, J.R. & CRESSWELL, P. (1987). NK susceptibility varies inversly with target cell class I HLA antigen expression. *J. Immunol.*, **138**, 1657.

SZOLLOSI, J., DAMJANOVICH, S., GOLDMAN, C.K. & 6 others (1987). Flow cytometric resonance energy transfer measurements support the association of a 95-kDa peptide termed T27 with the 55 kDa Tac peptide. *Proc. Natl Acad. Sci. USA*, **84**, 7246.

TANIGUCHI, K., PETERSON, M., HOGLUND, P., KIESSLING, R., KLEIN, G. & KARRE, K. (1987). Interferon gamma induces lung colonization by intravenously inoculated B16 melanoma cells in parallel with enhanced expression of class I major histocompatibility complex antigens. *Proc. Natl Acad. Sci. USA*, **84**, 3405.

YTHIER, A. & HERCEND, T. (1986). Subsets of human peripheral blood natural killer cells. In *Progress in Immunology, VI*, Cinader, B. & Miller, R.G. (eds) p. 935. Academic Press: New York.

ZOON, K.C., KARAZAKI, Y., ZUR NEDDEN, D.L., HU, R. & ARNHEITER, H. (1986). Modulation of epidermal growth factor receptors by human α interferon. *Proc. Natl Acad. Sci. USA*, **83**, 8226.